# Synthesis of Antifungal Agents from Xanthene and Thiazine Dyes and Analysis of Their Effects

**DOI:** 10.3390/nano6120243

**Published:** 2016-12-20

**Authors:** Joo Ran Kim, Stephen Michielsen

**Affiliations:** 1 Fiber Science & Apparel Design, Cornell University, Ithaca, NY 14850, USA; jk992@cornell.edu; 2 Textile Engineering, Chemistry and Science, North Carolina State University, 2401 Research Dr., Raleigh, NC 27695, USA

**Keywords:** antifungal photosensitizer, nanofiber, *Aspergillus*, *Chaetomium*, *Magnaporthe*

## Abstract

Indoor fungi growth is an increasing home health problem as our homes are more tightly sealed. One thing that limits durability of the antifungal agents is the scarcity of reactive sites on many surfaces to attach these agents. In order to increase graft yield of photosensitizers to the fabrics, poly(acrylic acid-*co*-styrene sulfonic acid-*co*-vinyl benzyl rose bengal or phloxine B) were polymerized and then grafted to electrospun fabrics. In an alternative process, azure A or toluidine blue O were grafted to poly(acrylic acid), which was subsequently grafted to nanofiber-based and microfiber-based fabrics. The fabrics grafted with photosensitizers induced antifungal effects on all seven types of fungi in the order of rose bengal > phloxine B > toluidine blue O > azure A, which follows the quantum yield production of singlet oxygen for these photoactive dyes. Their inhibition rates for inactivating fungal spores decreased in the order of *P. cinnamomi*, *T. viride*, *A. niger*, *A. fumigatus*, *C. globosum*, *P. funiculosum*, and *M. grisea*, which is associated with lipid composition in membrane and the morphology of fungal spores. The antifungal activity was also correlated with the surface area of fabric types which grafted the photosensitizer covalently on the surface as determined by the bound color strength.

## 1. Introduction

Antimicrobial fabrics based on natural or synthetic polymers treated with silver ions and copper nanoparticle have been used often [1,2]. Recently, 4-amide-piperidine-C12 hydrogel coated silicone films exhibited antimicrobial effects on both Gram-negative, Gram-positive bacteria and fungi [3]. Poly(methyl methacrylate) coated poly(2-hydroxyethyl methacrylate) films had been reported to have an antimicrobial effect on *Candida albicans* for up to 28 days [4]. However, these grafted chemicals have been reported to produce adverse effects on humans, resulting in an accumulation in the human skin. Silver can diffuse into the skin, darken upon exposure to sunlight, resulting in a blue or gray discoloration of the skin [5]. Hence, non-hazardous dye materials such as photosensitizers (PS) have been investigated intensively for the replacement of silver and copper, and hazardous chemicals. In particular, xanthene dyes such as rose bengal, phloxine B, and erythrosine have been shown to exhibit antimicrobial activity and are considered safe [6], since they are commonly used as dyes in the food, cosmetics and textiles where they are approved as safe color additives in the USA, the European Union (EU) and Japan [7]. Furthermore, they are less expensive, have better coloring, and are easier to produce than natural dyes [8]. Methylene blue and toluidine blue O have also been commonly used as safe PS [9]. Azure A and Azure A eosinate have been reported to inhibit 60% of the growth inhibition of *C. albicans*. Furthermore, various PS have been shown to be effective against fungi and yeast such as *Aspergillus fumigatus* and *Kluyveromyces fragilis*, *Kluyveromyces marxianus* and *C. albicans* [10]. Hong and Gang have studied antimicrobial properties using rose bengal incorporated into polyurethane-coated leather by a painting method [11]. However, there are fewer studies using PS grafted to polymeric materials. These limitations have affected the utilization of photodynamic actions in various applications. 

Photodynamic therapy (PDT) is a treatment with PS, which can be activated by exposure to visible light in a specific wavelength range. PS has a stable electronic configuration, which is in a singlet state in their lowest or ground energy level, S_0_ which means that there are no unpaired electron spins [12]. Upon absorption of a photon within a specific wavelength range, a molecule is promoted to an excited state, S_1_ or S_2_, with higher energy [13]. The exited singlet state of PS can return to the ground state by emitting a photon as light energy, i.e., by fluorescence. Alternatively, the molecule may convert to the triplet state, T_1_ or T_2_, via intersystem crossing, which involves a change in the spin of an electron [14]. The triplet ground state of oxygen exchanges spin with the triplet excited state of the dye to return the dye to its singlet ground state and to produce singlet oxygen at the same time. 

Singlet oxygen (^1^O_2_) is the first excited electronic energy state of molecular oxygen species. It is one of the most active intermediates involved in chemical and biochemical reactions. Hence, ^1^O_2_ can react with many kinds of biological molecules, such as DNA, proteins and lipids, resulting in chemical reactions due to the production of this reactive oxygen species [15].

*Aspergillus niger*, *Aspergillus fumigatus*, *Trichoderma viride*, *Penicillium funiculosum*, and *Chaetomium globosum* are the most common indoor fungal species that are opportunistic human pathogens resulting in aspergillosis and pneumocytosis in immunosuppressed patients and allergy, rhinitis in healthy humans [16,17]. *Magnaporthe grisea* and *Phytophthora cinnamomi* (Oomycetes) are plant pathogens inducing, respectively, rice blast resulting in serious crop loss globally [18] and root rot and canker on *Eucalyptus* [19].

In this study, photosensitizers rose bengal (RB), phloxine B (PB), azure A (AA), and toluidine blue O (TBO), were either copolymerized with acrylic acid or grafted to poly(acrylic acid), and then grafted to fabrics consisting of nanofiber-based fabrics or microfiber-based fabrics. The antifungal effects of the fabrics were then evaluated against seven types of fungi and analyzed antifungal effect.

## 2. Materials and Methods

### 2.1. Materials

Poly(acrylic acid) (PAA, average *M*_V_ ~450,000), 4-vinyl-benzyl chloride, 4-styrene sulfonic acid, acrylic acid (AC), nylon 6,6 pellets, formic acid (reagent grade > 95%), phloxine B (PB), azure A (AA), toluidine blue O (TBO), potato dextrose agar (PDA), potato dextrose broth, phosphate buffer saline (pH 7.0), Tween^®^ 20, RPMI 1640 medium, sterile water, 3-(*N*-morpholino)-propanesulfonic acid (MOPS), and 4-(4,6-dimethoxy-1,3,5-triazin-2-yl)-4-methylmorpholinium chloride (DMTMM) were purchased from Sigma Aldrich Chemical Co. (St. Louis, MO, USA). RB and meltspun nylon 6.6, Cerex Spectramax^®^ were donated by LaamScience (Cary, NC, USA), while 0.5 McFarland standard for turbidity measurements was purchased from Fisher Scientific Co. (Pittsburgh, PA, USA). *Aspergillus niger* (ATCC 6275), *Trichiderma viride* (ATCC 28020), *Penicillium funiculosum* (ATCC 10509), *Aspergillus fumigatus* (ATCC 13073) and *Chaetomium globosum* (ATCC 6205) were donated from the United States Department of Agriculture, Agricultural Research Service, Peoria, IL, USA. *Magnaporthe grisea* and *Phytophthora cinnamomi* (fungus-like organism) were donated from the North Carolina State University, Plant Pathogens Department, Raleigh, NC, USA.

### 2.2. Preparation of Nanofiber-Based Fabrics

**Figure 1:**
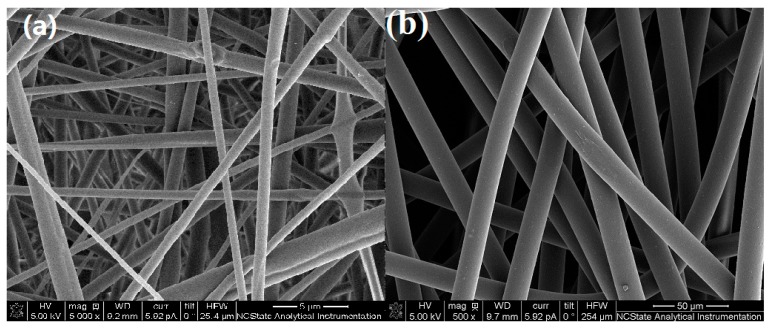
Nanofibers using electrospinning using roller collector, (**a**) nylon 6,6 fabric consisting of nanofibers with average diameter 505 nm (σ = 152.5 nm); and (**b**) melt spun microfibers (Cerex Spectramax^®^ nylon 6,6) with diameter 17.3 µm (σ = 0.86 µm).

Nanofiber-based fabric was prepared using electrospinning, which produced fibers in the range from 200 nm to 5 µm in diameter as shown Figure 1a. A high voltage (20 kV) was applied between needle tip and roller collector. First, 18% *w*/*v* nylon 6,6 pellets were dissolved into formic acid and agitated at 70 °C for 8 h. The solution was put into a 10-mL syringe and then placed on the pump. The feeding rate of solution was 1 mL per hour. 

### 2.3. Preparation of Water-Soluble Photosensitizers

**Figure 2:**
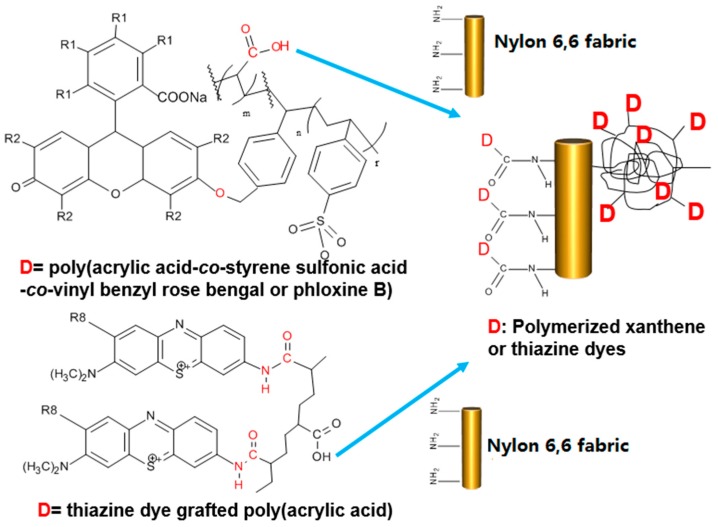
The grafting scheme of poly(acrylic acid-co-styrene sulfonic acid-co-vinyl benzyl rose bengal or phloxine B) or thiazine dyes grafted with poly(acrylic acid) (PAA) to the nylon fiber forming random coil shape. D is polymerized dye molecule such as polymerized xanthene dyes or thiazine dyes grafted with PAA. For RB, R_1_ and R_2_ are I and Cl. For PB, R_1_ and R_2_ are Br and Cl and TBO has methyl group at R_8_. Rose Bengal = RB; phloxine B = PB; toluidine blue O = TBO.

The xanthene dyes (RB or PB) were incorporated into poly(acrylic acid) (PAA) via polymerization. Briefly, 3 g of RB or 2.5 g of PB was stirred in a 70-mL solution of 50:50 *v*/*v* distilled water/acetone and 0.51 mL of 4-vinyl-benzyl chloride at 65 °C for 3 h. The precipitate, vinyl benzyl-xanthene dye (VBXD where XD refers to the xanthene dye RB or PB), was filtered and washed. Next the VBXD was dissolved in water and acetone at the volume ratio of 1:1 and polymerized with 4-styrene sulfonic acid (SAA) and acrylic acid (AC) at 1:40:140 molar ratio of VBXD: SSA:AC to increase water solubility to the polymers and to confer functional groups with which to graft to nylon [20,21]. After copolymerization, poly(acrylic acid-*co*-styrene sulfonic acid-*co*-vinyl benzyl rose bengal or phloxine B) were obtained. A condensing agent, 4-(4,6-dimethoxy-1,3,5-triazin-2-yl)-4-methylmorpholinium chloride (DMTMM), was used to promote chemical reactions between –COOH groups of PAA of polymerized dye solution to NH_2_ end-groups of nylon 6,6 fabrics to produce amide linkages as shown in Figure 2.

The thiazine dyes were also grafted to PAA as follows. A 5% *w*/*v* PAA solution in distilled water was prepared at room temperature; 100 µmol/L of AA or TBO was added to the PAA solution. After stirring for 1 h, 0.3 g DMTMM was added to the mixture [22]. After stirring for another 3 h, the thiazine dye-grafted-PAA solution was produced as shown in Figure 2. 

### 2.4. Grafting of Photosensitizers to Fabrics

In order to graft the photosensitizer containing polymers to the fabric, each polymerized solution was prepared at 100 µmol/L concentration in a flat-bottomed dish, which had been covered with aluminum foil to exclude light. Electrospun nanofiber-based fabrics and microfiber-based fabrics were immersed into the solution bath. The condensing agent, 0.3 g DMTMM, was dissolved in the solution bath. After 12 h, the fabric was removed from the solution and placed into an oven (Werner Mathis AG LTF 134489, Concord, NC, USA) for 1 min at the temperature of 170 °C. 

### 2.5. Preparation of Inoculum

*A. fumigatus*, *A. niger*, *T. viride*, and *P. funiculosum*, *C. globosum*, *P. cinnamomi* and *M. grisea* were cultured at 35 °C on potato dextrose agar (PDA) plates for seven days. Broth medium, RPMI-1640 which contains 0.2% glucose and 0.165 mol/L MOPS (3-*N*-morpholino propanesulfonic acid) at pH 7.0, was used as medium for five fungi, while the potato dextrose broth (PDB) was used as medium for *P. cinnamomi* and *M. grisea*. Next, 10 mL broth medium with 0.01% wetting agent Tween^®^ 20 was added to the cultures and then scraped to separate spores. The scraped solution in the culture was filtered through Millipore^®^ polytetrafluoroethylene film to remove hyphae. 

### 2.6. Inhibition Zone Test

The inhibition zone test of fabrics grafted with photosensitizers RB, PB, TBO and AA was performed as follows. A 100 µL inoculum of each strain prepared above at 2 × 10^6^ CFU/mL was transferred to a PDA plate and then a 5 × 5 cm piece of the fabrics prepared above were placed onto the inoculated PDA plate. The plates were placed into a tray and the tray was placed under the lamp. A water-filled pyrex glass dish was placed between the tray and the lamp to absorb infrared light from a photoflood lamp (Smith Victor, Griffith, IN, USA) to avoid heating the tray. The lamp was placed 35 cm above the glass dish and the inoculated PDA plates were placed below the glass dish. The light intensity was measured using a digital illuminance meter (Model LX1330B, Union City, CA, USA) at 15,500 Lux. The plates were kept under this exposure for 5 h at 24 °C and then incubated for seven days in the dark at 35 °C. 

### 2.7. Minimum Inhibitory Concentration—Broth Microdilution Test

In order to test the effectiveness of the free photosensitizer dyes, minimum inhibitory concentration (MIC) was conducted according to the CLSI M38-A standard [23]; 100 μL of 500 µmol/L aqueous solutions of RB, PB, AA, and TBO solutions were transferred to the first wells in the 96-well plates. Then each column was diluted by two-fold up to 0.98 µmol/L. The spore concentration was adjusted to lie in the range of 2 × 10^6^ CFU/mL using 0.5 McFarland standard as a reference and a hemocytometer (Hausser Bright-Line and Hylite Counting Chambers, Horsham, PA, USA). Then 100 µL inoculum of fungal spores was deposited into each well containing the free photosensitizer solutions prepared above. The 96-well plates were then illuminated for 3 h at 24 °C. Finally, a 96-well plate was incubated at 35 °C for all fungi for 48 h. All tests were conducted three times and then MIC was selected as the highest concentration.

### 2.8. Quantitative Antifungal Assay

A quantitative antifungal assay was also conducted following a modified ASTM E2149-01 method [24]. The inoculum was diluted into sterile phosphate buffer solution (pH 7.0) to obtain a final concentration of 2 × 10^5^ CFU/mL. The prepared inoculum (50 mL) was added into each 125 mL Erlenmeyer flask and then treated or untreated circular fabric samples with 4.8 cm in diameter were immersed in the flask, which was then covered with a cap with a hole in it. The flasks were placed under the light as above for 3 h at 24 °C and the flask was shaken at 200 strokes/minute throughout the test. Then a100 µL aliquot of inoculum from each flask was transferred to individual wells of a 96-well tray every 30 min; 96-well trays were incubated in the dark at 35 °C for 48 h. The optical density (OD) was measured at 405 nm using Biotek^®^ Synergy HT multi-mode reader. The ODs of the treated fabric with inoculum (*B*) was subtracted from the ODs of the untreated fabric (*A*) with inoculum [24,25]. The final reduction percentage was calculated as follows:

(1) $$\frac{A-B}{A}\times 100$$

where *A* is the OD at 405 nm of the solution in the well containing the inoculum exposed to the untreated fabric sample and *B* is the OD of the solution in the well corresponding to each treated fabric after the specified contact time between the treated and illuminated fabric sample. 

### 2.9. Characteristics

The specific surface areas of untreated nanofiber-based fabric and microfiber-based fabrics were obtained from the Brunauer-Emmett-Teller (BET) analysis using liquid nitrogen as the adsorbate and a Gemini VII 2390p physiosorption analyzer from Micrometrics coupled with SmartPrep 065 degassing unit (Micromeritics Instrument Co., Norcross, GA, USA). The chopped fabric sample (5 × 5 cm) consisting of nanofibers (0.109 g) or microfibers (0.249 g) was used for BET analysis. Field emission scanning electron microscopy (JEOL 6400) (JEOL USA Inc., Peabody, MA, USA) was used to examine the morphology of spores from the seven pathogens and to examine the nylon fibers. The amounts of RB, PB, AA and TBO grafted to the nanofiber-based and microfiber-based fabrics were obtained from reflectance measurements over the spectral range 360–700 nm using a Datacolor SF600X (Lawrenceville, NJ, USA) spectrometer. The fabrics were folded to ensure optical opacity, which required at least 16 layers thick for microfiber-based fabrics and at least five layers for nanofiber-based fabrics. Each measurement was repeated two times in four different spots of the samples. 

## 3. Results and Discussion

Antifungal agents must be on the surface of fibers in order to be able to inhibit the microbes. However, typical treatments with antifungal agents often wash off easily. Thus, it is advantageous to permanently attach the antifungal agents. The amino groups of nylon 6,6 can react with carboxylic acid groups from the polymerized, PAA-based photosensitizer dyes. This enabled the permanent grafting of the photosensitizer dyes that had been either copolymerized with AC-(xanthene dyes) or that had been grafted onto PAA-(thiazine dyes), thus producing amide linkages.

### 3.1. Specific Surface Area of Fabrics and Color Strength

Figure 1a,b shows the SEM images of the electrospun nanofibers and the melt-blown microfibers. The diameters of the fibers were measured from the SEM images. The diameters of two types of fabrics were calculated by randomly selecting 30 microfibers and more than 100 nanofibers under the SEM images. The average diameter of the nanofibers was 505 nm (σ = 152 nm), while microfibers had an average diameter of 17.3 µm (σ = 0.86 µm). The specific surface area was measured directly using BET analysis, giving a specific surface area of 28.8 m^2^/g for the nanofiber-based fabric, while the microfiber-based fabric had a specific surface area of 1.41 m^2^/g. Thus, the BET analysis gives a specific surface area ratio of 20:1 for the nanofiber-based fabrics to the microfiber-based fabrics. 

The amount of photosensitizer dye on the fabric surface should be proportional to the color strength of the fabric. The fabric color strength was calculated using reflectance percentage and *K*/*S* value as shown in Equation (2) [26].

(2) $$\frac{K}{S}=\frac{{\left(1-R_{\infty }\right)}^{2}}{2R_{\infty }}=F\left(R_{\infty }\right)$$

where *K* and *S* are absorbance and scattering coefficients (cm^−1^), and *R_∞_* is the reflectance for infinite thickness. If the fabric is sufficiently thick so that it is opaque, *R_λ_* = *R_∞_*, where the reflectance is measured at the wavelength of maximum absorbance [27].

The *K*/*S* values were converted to relative color strength (%) using Equation (3) [28].

(3) $$\mathrm{Relative}\;\mathrm{Color}\;\mathrm{Strength}\;\left(\%\right)\;=\frac{K/S\;\mathrm{of}\;\mathrm{treated}\;\mathrm{fabric}}{K/S\;\mathrm{of}\;\mathrm{untreated}\;\mathrm{fabric}}\times 100$$

Table 1 gives both the *K*/*S* value and the color strengths for each of the fabrics used in this study. The results in Table 1 imply that there is 6.6 to 12 times more relative color strength or more amount of dye on the nanofiber-based fabrics than on the microfiber-based fabrics.

### 3.2. Antifungal Activity—Minimum Inhibitory Concentration (MIC) Test

Before testing the antifungal activities of fabrics with the photosensitizer dyes attached, the antifungal effectiveness of the free dyes was tested using the MIC test. The results shown in Table 2 indicate that for RB there was no visible turbidity or growth at 62.5 µmol/L while concentrations below 62.5 µmol/L showed visible hyphal growth on *A. niger* and *A. fumigatus*, *P. funiculosum*, and *C. globosum*. For *T. viride*, RB displayed the MIC of 31.2 µmol/L and 15.6 µmol/L for *P. cinnamomi*. For PB, *P. funiculosum*, and *C. globosum* showed the largest resistance with MIC at 125 µmol/L, while *A. niger* and *A. fumigatus* had MIC at 62.5 µmol/L. TBO and AA both had much higher MICs than RB or PB. TBO has an MIC of 62.5 µmol/L on *A. niger*, *A. fumigatus*, and *T. viride*. For AA, below 125 µmol/L visible growth and turbidity was observed for *A. niger* and *A. fumigatus*, *P. funiculosum* and *C. globosum*, but not *T. viride*, giving MIC of 62.5 µmol/L. RB, PB, TBO. AA showed the lowest MIC on *T. viride* while exhibiting the largest MIC on *P. funiculosum* or *C. globosum*.

For *P. cinnamomi*, the MIC was very low, with an MIC of 15.6 (RB) and 31.25 (PB) µmol/L. However, *M. grisea* was quite resistant to inhibition with an MIC of 125 µmol/L for RB while PB inhibited *M. grisea* at 250 µmol/L. AA and TBO inhibited fungal growth of *M. grisea* above 250 µmol/L and *P. cinnamomi* at 62.5 µmol/L. *M. grisea* showed the largest resistance to photosensitizers showing the highest MIC at 250 µmol/L.

### 3.3. Antifungal Activity—Inhibition Zone Test

To evaluate the antifungal effectiveness of the surface-bond photosensitizers, two types of antifungal assays were conducted against seven fungi types. The first method examined visible growth of hyphae and germination on the fabric to identify the zone of inhibition. This test is a qualitative test that relies on the ability of the antifungal agent to diffuse away from the treated fabric in combination with the antifungal activity of the agent. Large inhibition zones indicate that the agent is quite mobile while small inhibition zones indicate either little mobility or a lack of effectiveness.

**Figure 3:**
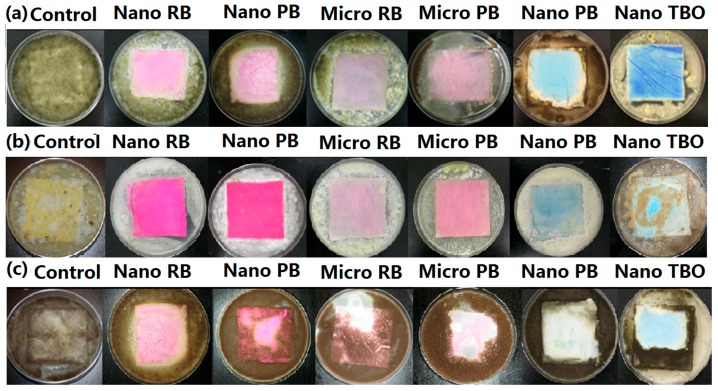
The inhibition zone test of nano and micro fabric grafted with RB, PB, AA and TBO on (**a**) *P. cinnamomi*; (**b**) *T. viride*; and (**c**) *M. grisea*, The 1st column is the control, the 2nd is the nano fabric grafted with RB, the 3rd is the nano fabric grafted with PB, the 4th is the micro fabric grafted with RB, the 5th is the micro fabric grafted PB, the 6th is the nano fabric grafted with TBO, the 7th is the nano fabric grafted with azure A (AA).

Figure 3 shows inhibition zones for (**a**) *P. cinnamomi*; (**b**) *T. viride*; and (**c**) *M. grisea* on nanofiber-based and microfiber-based fabrics. In general, the fabrics exhibited less growth of *P. cinnamomi* and *T. viride* on the surface, compared to the growth of *M. grisea*. The nanofiber-based fabrics grafted with RB and PB showed no growth of *P. cinnamomi* or *T. viride* on the fabric, but nanofiber-based fabrics grafted with AA showed *P. cinnamomi* growth around the edge of the fabric and substantial growth of *T. viride* on the fabric. The microfiber-based fabric with RB and PB showed little *P. cinnamomi* and *T. viride* colony growth on the fabric while the control was completely covered by growth and germination. Most microfiber-based fabrics grafted with AA and TBO exhibited no inhibition of fungal growth (not shown). On the other hand, *M. grisea* exhibited substantial growth on all fabrics except for the RB-grafted nanofiber-based fabric. Figure 3 also shows that the zone of inhibition for all treated nanofiber-based and microfiber-based fabrics was negligible although there was very little fungi growth on the fabrics treated with RB or PB. Even though TBO and AA were less effective, the zone of inhibition was still negligible. This is indirect evidence that the antifungal photosensitizers were indeed grafted to the fabric surfaces. The inhibition zone is expected to be nearly zero if the photoactive polymer is covalently attached to the fabric since the active antifungal agent is believed to be singlet oxygen, which has a short lifetime, limiting diffusion to around 0.1 mm [29] and thus limiting antifungal activity to close proximity to the polymerized photosensitizers.

### 3.4. Antifungal Activity—Quantitative Assessment

The ability of the dyes RB, PB, TBO, and AA immobilized on the fabrics was investigated as functions of the photo-irradiation time, up to a maximum of 180 min. All the fabrics grafted with RB, PB, AA, and TBO reduced growth of all seven types of pathogens as illustrated by the reduction of the ODs. The largest inhibition among the human pathogens tested in this study was on *T. viride*, while the OD reduction of *C. globosum* and *P. funiculosum* was much less than for the others. The plant pathogens, *M. grisea*, displayed the highest resistance to all types of photosensitizers, while *P. cinnamomi* showed the least resistance to fabrics grafted with each of the dyes. In order to compare inhibition quantitatively among various variables, the four types of photosensitizers and all seven types of pathogens, we analyzed the inhibition rates and fitted them using Equation (4) developed by Kim and Michielsen [30].

ln*OD*_t_ = ln*OD*_0min_ – *k*_S_[*D*_0_]*t*exp(−*k*_D_*t*) (4)

where *OD*_t_ is the optical density of the fungal suspensions at 405 nm. These fungal suspensions were obtained by growing the fungi that survived after t min exposure to the treated fabrics under illumination, as described in the experimental section; *k*_s_ is the inhibition rate of the growth, *D*_0_ is the initial dye concentration, *t* is the exposure time, and *k*_D_ is the reduction rate of concentration of dye by photobleaching.

Equation (4) was used to compare the inhibition rate of each fabric grafted with RB, PB, TBO and AA against seven pathogens and the photobleaching rate of the grafted dyes. The significance of the data was evaluated using the student *t*-test and ANOVA.

**Figure 4:**
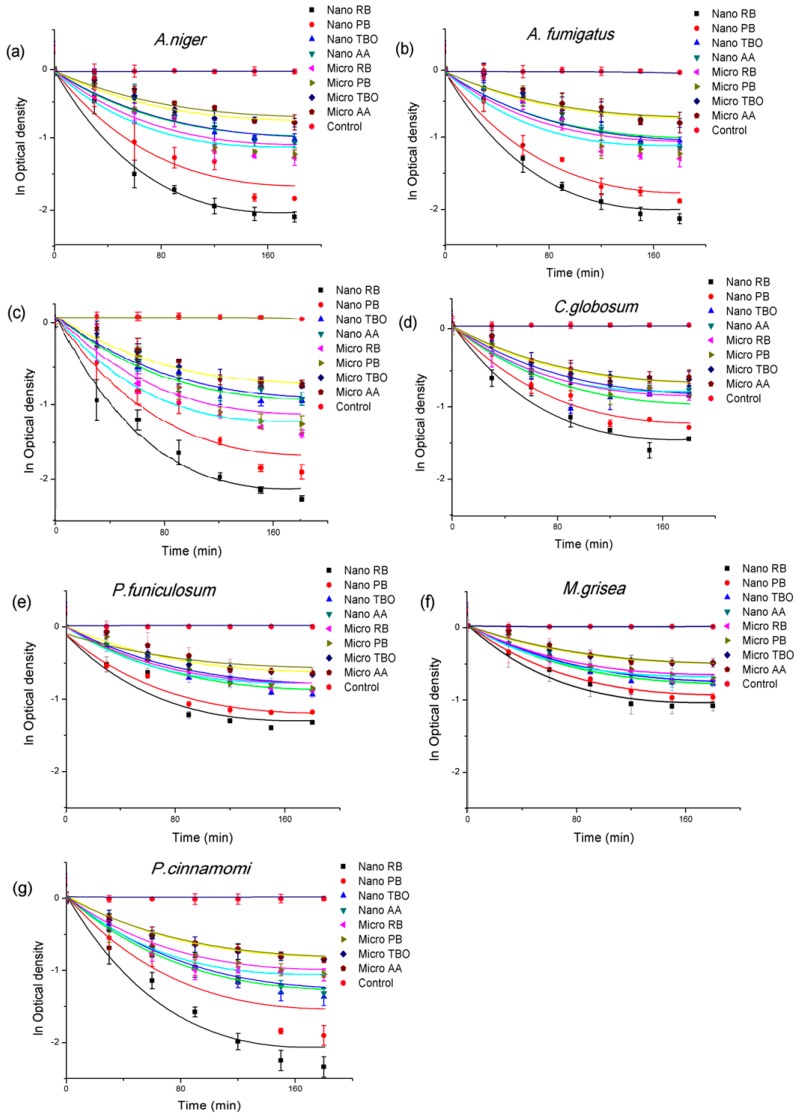
The optical density (OD) reduction by nano and micro fabrics grafted with RB, PB, TBO, and AA graphed as a function of time under illumination on (**a**) *A. niger*; (**b**) *A. fumigatus*; (**c**) *T. viride*; (**d**) *C. globosum*; (**e**) *P. funiculosum*; (**f**) *M. grisea*; and (**g**) *P. cinnamomi*. Note: all axes are to the same scale to aid comparisons between materials.

The nanofiber-based fabric grafted with RB exhibited the largest OD reduction on *P. cinnamomi* and *T. viride* with the inhibition rates of 3.7 × 10^−2^ and 3.5 × 10^−2^ L/µmol·min, respectively. *M. grisea* had the lowest inhibition rate of 1.4 × 10^−2^ L/µmol·min for the RB-treated fabric. The next highest inhibition rates were seen with PB, showing 2.5 × 10^−2^ and 2.4 × 10^−2^ L/µmol·min against *P. cinnamomi* and *T. viride*. In general, the inhibition rates for the treated fabrics decreased in the order RB > PB > TBO > AA. Likewise, the inhibition rates for the fungi decreased in the order *P. cinnamomi* > *T. viride* > *A. niger* > *A. fumigatus* > *C. globosum* > *P. funiculosum* > *M. grisea*. The results also show that the inhibition rate for the nanofiber-based fabrics was always higher than for the microfiber-based fabrics, as shown in Figure 4.

**Figure 5:**
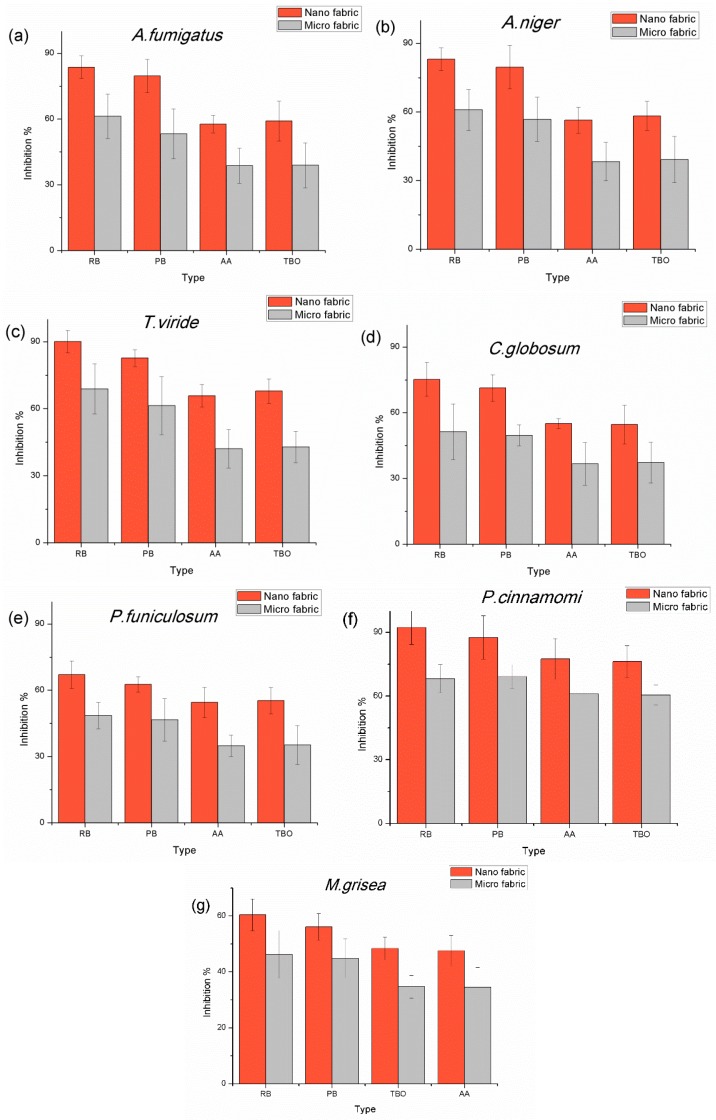
Inhibition percentages of the nano and micro fabrics with immobilized RB, PB, AA, and TBO photosensitizers on (**a**) *A. fumigatus*; (**b**) *A. niger*; (**c**) *T. viride*; (**d**) *C. globosum*; (**e**) *P. funiculosum*; (**f**) *P. cinnamomi*; and (**g**) *M. grisea*.

Figure 5 shows the final inhibition percentage and compares the inhibition percentage of nanofiber-based fabrics with microfiber-based fabrics grafted with photosensitizers. All nanofiber-based fabrics grafted with photosensitizers showed higher inhibition than the microfiber-based fabrics. The average inhibition % in nanofiber-based fabrics was over 70% inhibition, while microfiber-based fabrics gave less than 50% inhibition. Finally, the results showed the increasing inhibition percentage in the order of AA < TBO < PB < RB on the grafted fabrics. The fabrics grafted with AA and TBO showed similar effect but the fabrics grafted with TBO showed slightly higher inhibition rates; however, the fabrics grafted with RB showed much higher inhibition rates than the fabrics grafted with PB. On average, the inhibition rate for the nanofiber-based fabrics was 1.5 times higher than on the microfiber-based fabrics with the same photosensitizer. 

### 3.5. Morphology of Fungal Spore

**Figure 6:**
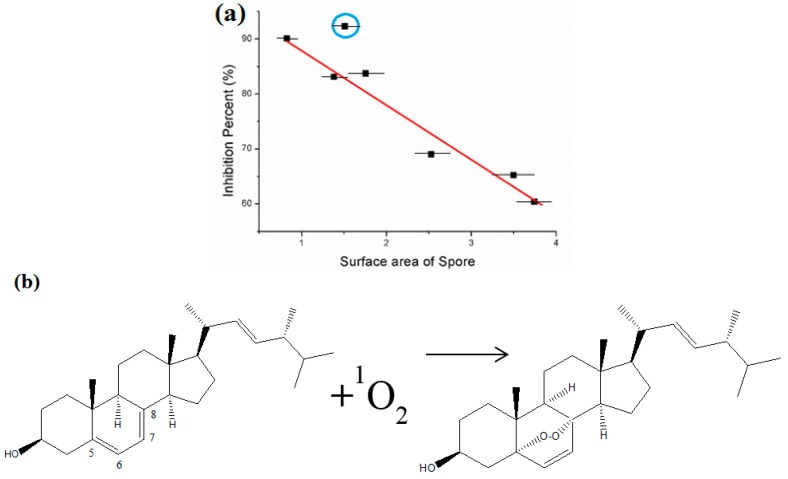

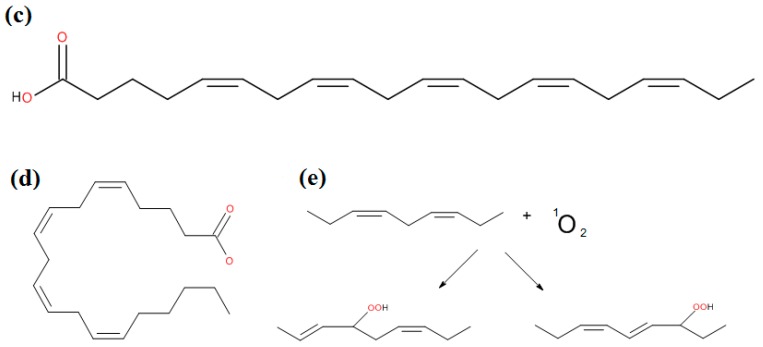
The relationship between antimicrobial activity and properties of microorganism: (**a**) the graph of the inhibition percent and surface area of spores; (**b**) photooxidation of ergosterol to ergoperoxide in ascomycota fungal membrane [34]; (**c**) eicosapentaenoic acid (EPA); (**d**) arachidonic acid (AR); (**e**) reactions on double bonds by singlet oxygen in unsaturated fatty acid chains.

Using SEM images, the sizes of fungal spores were measured from 20 to 50 randomly selected spores. The shape of conidia of *A. niger* and *A. fumigatus* are spherical and present individually or as strings, and the diameters were 2.4 µm (σ = 0.34) and 2.7 (σ = 0.58), respectively. The average diameters of *T. viride* and *P. cinnamomi* spores are 1.8 µm (σ = 0.28) and 2.5 (σ = 0.43). Conidia of *P. funiculosum* are 3.2 µm (σ = 0.52) with globule shapes. *C. globosum* and *M. grisea* spores have larger diameters of 3.7 µm (σ = 0.46) and 3.8 (σ = 0.27), as shown in Figure 6. Hence, the antifungal agents were less effective on *M. grisea*, which has a larger size than *T. viride*. Prokaryotic bacteria appear to be inhibited in a single-hit process by PDT. This occurs at much lower concentration of photosensitizers than for fungi because of their lack of a nuclear membrane [31]. It has been shown that *C. albicans* is more difficult to inhibit by PDT, requiring a higher concentration at around 2–5 mg/mL of TBO and stronger light doses because eukaryotic fungus is more complex and a larger organism, requiring multiple hits to induce cell death [32]. The largest size, *M. grisea* requires higher dye concentration to be inhibited because bigger spore size requires a multi-hit process by singlet oxygen. The photosensitizer cannot easily reach inside spores to inhibit it. 

The six types of ascomycota fungi follow a linear relationship between surface area of their spores and the inhibition percent, as shown in Figure 6a. The inhibition percent decreases with increasing surface area of spores. However, *P. cinnamomi* seems to be an outlier. It shows more antifungal activity as compared to its size. This is because the initial attack of singlet oxygen occurs mainly on lipids, especially at the carbon–carbon double bonds in unsaturated fatty acids in biological molecules to form hydroperoxides [33]. ^1^O_2_ is very electrophilic, which is capable of directly oxidizing electron-rich double bonds in cells, as shown in Figure 6b,e [14,33,34]. *P. cinnamomi* has been reported to include the high amount of arachidonic acid (AR) and eicosapentaenoic acid (EPA) (see chemical structures in Figure 6d,e [35]. The presence of double bonds of long unsaturated fatty acids, such as 5,8,11,14,17-EPA is a good target for singlet oxygen to inhibit membrane function of fungi. *P. cinnamomi* has high contents of EPA and AR instead of ergosterol [36]. Thus *P. cinnamomi* can be easily attacked by singlet oxygen due to its high amount of polyunsaturated fatty acids in the membrane, thus forming hydroperoxides in the cell membrane in order to inhibit cell functions [37].

## 4. Conclusions

In order to increase graft yield of photosensitizers on the surface of fabrics, poly(acrylic acid-*co*-styrene sulfonic acid-*co*-vinyl benzyl RB or PB) were polymerized and AA or TBO was polymerized with poly(acrylic acid). All four types of copolymers were successfully grafted to microsize- or nanosize-based fabrics. The inhibition zone test showed that the nanofibers grafted with RB and PB displayed no growth on the fabrics, but micro fabrics grafted with AA and TBO had little effectiveness against the seven types of fungi used in this study. The quantitative method was also used to compare inhibition rate for the different photosensitizers against the different fungi.

In the results, nano fabrics grafted with RB resulted in up to a 92% reduction in growth with a high inhibition rate. Nano fabric grafted with RB had the largest inhibition rate (*k*_s_) on *P. cinnamomi*, up to 3.7 × 10^−2^ and 3.5 × 10^−2^ L/µmol·min, while on *M. grisea* exhibited the lowest inhibition rate of 1.4 × 10^−2^ L/µmol·min. This activity was related to fungal morphologies and lipid composition of the cell membranes. *P. cinnamomi* had a high amount of arachidonic acid and eicosapentaenoic acid, which were good targets for photooxidation by singlet oxygen, while filamentous ascomycota fungi had ergosterol instead. *M. grisea* had the largest spore size with diameters of 3.9 µm and exhibited the highest resistance to singlet oxygen due to inhibition by a multi-hit process with singlet oxygen. Finally, it was concluded that the results showed the increasing inhibition percentage in the order of AA < TBO < PB < RB. The nano fabrics had higher color strength of photosensitizers and were more effective on fungi because of a larger surface area at 29 m^2^/g, while micro fabrics had 1.4 m^2^/g. The inhibition rates for RB, PB, TBO and AA for all fabrics had efficiency in the decreasing order of *P. cinnamomi*, *T. viride*, *A. niger*, *A. fumigatus*, *C. globosum*, *P. funiculosum*, and *M. grisea*, which was associated to lipid composition in membrane and morphology of fungal spores. 

## Figures and Tables

**Table 1:** Reflectance percentages, *K/S* values and color strength (%) of nano and micro fabrics grafted with RB, PB, TBO, and AA.

Grafting Dye	Reflectance % at λ_max_		*F*(*R*_∞_)		Color Strength (%)	
	Nano	Micro	Nano	Micro	Nano	Micro
RB	2.93 ± 0.13	21.2 ± 0.86	0.16	0.015	60.1	5.0
PB	3.07 ± 0.21	20.7 ± 1.23	0.15	0.015	51.2	5.0
TBO	4.18 ± 0.19	21.5 ± 1.01	0.11	0.015	36.7	5.0
AA	4.94 ± 0.16	22.8 ± 0.97	0.09	0.014	30.7	4.7

**Table 2:** The minimum inhibition concentration (MIC) of RB, PB, AA, and TBO to prevent the fungal growth in broth microdilution tests by visual observation (the highest concentration to inhibit growth was selected among three tests).

Grafting Dye	MIC (µM)						
	*M. grisea*	*P. funiculosum*	*C. globosum*	*A. niger*	*A. fumigatus*	*T. viride*	*P. cinnmomi*
RB	125	62.5	62.5	62.5	62.5	31.2	15.6
PB	250	125	62.5	62.5	125	31.2	31.2
AA	250	125	62.5	125	62.5	62.5	62.5
TBO	250	125	125	62.5	62.5	62.5	62.5
